# Feedback and Direction Sources Influence Navigation Decision Making on Experienced Routes

**DOI:** 10.3389/fpsyg.2019.02104

**Published:** 2019-09-13

**Authors:** Yu Li, Weijia Li, Yingying Yang, Qi Wang

**Affiliations:** ^1^Department of Psychology, Sun Yat-sen University, Guangzhou, China; ^2^Department of Psychology, Montclair State University, Montclair, NJ, United States

**Keywords:** navigation, decision-making, navigation guidance, feedback, direction sources

## Abstract

When navigating in a new environment, it is typical for people to resort to external guidance such as Global Positioning System (GPS), or people. However, in the real world, even though navigators have learned the route, they may still prefer to travel with external guidance. We explored how the availability of feedback and the source of external guidance affect navigation decision-making on experienced routes in the presence of external guidance. In three experiments, participants navigated a simulated route three times and then verbally confirmed that they had learned it. They then traveled the same route again, accompanied with no, correct, or incorrect direction guidance, which latter two were provided by a GPS (Experiment 1), a stranger (Experiment 2), or a friend (Experiment 3). Half of the participants received immediate feedback on their navigation decisions, while the other half without feedback did not know if they had selected the correct directions. Generally, without feedback, participants relied on external guidance, regardless of the direction sources. Results also showed that participants trusted the GPS the most, but performed best with their friends as a direction source. With feedback, participants did not show differences in performance between the correct and incorrect guidance conditions, indicating that feedback plays a critical role in evaluating the reliability of external guidance. Our findings suggest that incorrect guidance without any feedback might disturb navigation decision-making, which was further moderated by the perceived credibility of direction sources. We discuss these results within the context of navigation decision-making theory and consider implications for wayfinding behaviors as a social activity.

## Introduction

Navigators have to gain sufficient experience to learn accurate landmarks or directions of a specific route in order to navigate freely and independently (Thorndyke and Hayes-Roth, 1982). Before that, when being uncertain about route directions, navigators may turn to external guidance. Navigational guidance takes many forms. Navigators now generally use Global Positioning System (GPS) devices to guide navigation. When GPS device is neither available nor helpful, people may ask another human, either a friend or stranger, for route directions. How do navigators believe in the external guidance? How do direction sources affect the trust of the direction givers? The present study explored the above questions by manipulating the availability of feedback and the source of directions to investigate their effects on spatial decision-making.

### Navigation Decision-Making With External Directional Guidance

Route knowledge primarily focuses on sequences of the turning directions and their reference locations, such as turning a particular direction at a specific intersection or landmark (“turning left at the second intersection”) (Brunyé et al., 2014). It is not a surprise that navigators would follow external navigation guidance, like a GPS, to complete wayfinding activities when they are uncertain about route directions. However, in some circumstances, navigators would like to have external guidance even though they have traveled through the route before. It is unclear what the navigator will follow when the external guidance and one’s route knowledge conflict.

Studies have shown that humans’ knowledge is susceptible to distortion from post-event information. Although the effect of post-event information on one’s knowledge has been investigated in various fields involving perceptual or semantic processing, few studies have examined it in the context of spatial decision-making. For instance, participants recalled seeing a yield sign instead of a stop sign at an intersection when post-event information replaced the original stop sign with a yield sign (Loftus and Hoffman, 1989). The fact that both semantic and perceptual representations can be distorted by post-event information has important implications for navigation decision-making (e.g., Roediger and McDermott, 1995; Slotnick and Schacter, 2004; Vannucci et al., 2012), as spatial knowledge usually involves semantic and sensorimotor representations (Wang et al., 2012, 2018). The present work aims to explore the effect of post-navigation information on spatial decision-making by presenting external directional guidance when navigators travel through experienced environments. If the route knowledge is robust, then navigators should be able to ignore incorrect external directions and instead adhere to their initial decisions. In contrast, if the route knowledge is relatively weak and not contextually connected to the broad navigated environment, then the external directional guidance, when incorrect, might interfere with navigation decisions.

The external directional guidance, when incorrect, could unconsciously interfere with spatial decision-making, as participants usually may not realize that the misleading information is exactly that, misleading. When people are less confident about their knowledge, they may also consciously make their navigation decisions based on the external guidance (Baron et al., 1996; Wright et al., 2010; Hirst and Echterhoff, 2012; Wright and Villalba, 2012; Goodwin et al., 2013; Cassidy et al., 2015; Hope and Gabbert, 2018). The current study explores two factors that may influence confidence in and reliance on one’s navigation decisions: post-decision feedback and direction source.

### The Effect of Post-decision Feedback on Navigation Decision-Making

Well-documented studies on testing effects have suggested that people benefit from feedback after having to retrieve relevant knowledge. Feedback after retrieving incorrect information, i.e., an indication of an error, reinforces the correct knowledge (e.g., Chan et al., 2006; Karpicke and Roediger, 2008; Chan, 2009; Thomas et al., 2010). While few studies have investigated how feedback affects spatial decision-making, testing effect research supports that spatial memory could benefit from having to recall (being tested on) route knowledge or spatial arrays of objects in certain situations (Carpenter and Pashler, 2007; Rohrer et al., 2010; Carpenter and Kelly, 2012; Wang et al., 2014; Kelly et al., 2015; Brunyé et al., 2019). For example, Kelly et al. (2015) found that feedback could benefit route learning when correctly retrieving spatial knowledge and before movement errors were made.

The present work extends the research on the effect of feedback to the domain of spatial decision-making. Specifically, we use route pictures to simulate the driving route, which did not have typical landmarks contained by a real environment. Although driving in a real world could involve landmarks, the external guidance usually involves route directions to assist spatial decision-making. In addition, for some environments with similar landmarks, such as a busy business district, or highways in the desert or farming area, navigators have to make navigation decisions primarily based on route directions.

**FIGURE 1:**
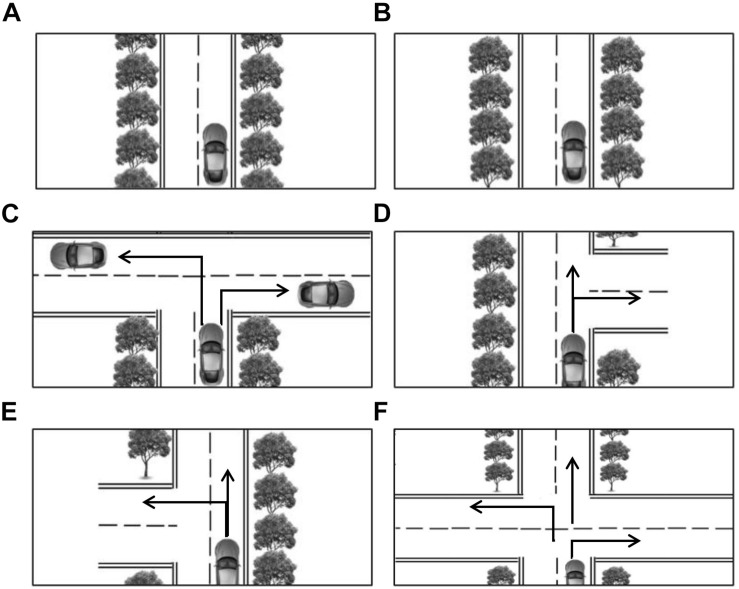
Road images used in Experiments. **(A,B)** Show straight routes; **(C–E)** illustrate T-junctions, and **(F)** shows a four-way intersection. The black arrows demonstrate possible driving directions, which are not displayed during formal navigation.

**FIGURE 2:**
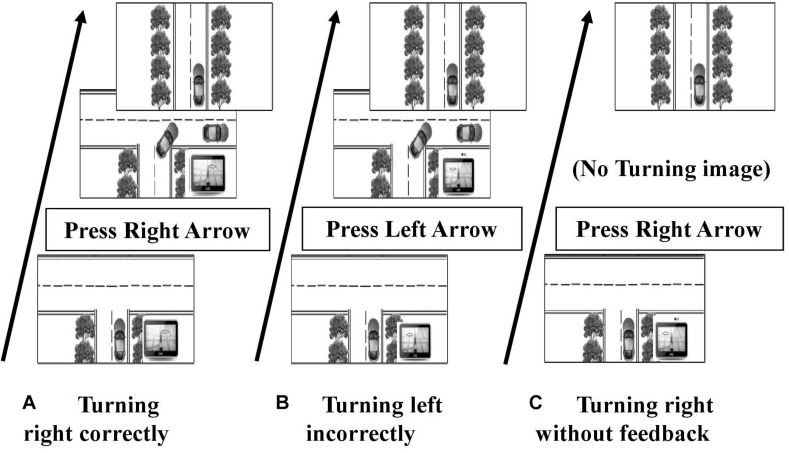
External guidance correctness and Turn feedback. If the correct direction for this intersection involves turning right, then the GPS showing to turn right is a correct direction **(A)**, while the GPS showing a left turn is an incorrect direction **(B)**. The turning image shows turning to correct direction at one intersection, regardless of the participant’s decision **(A,B)**. There would not be a turning image in the no-feedback condition **(C)**. A straight route is shown immediately after a turn at an intersection.

When traveling through a spatial environment, external directional guidance may remind the navigators that they have taken a wrong route. Otherwise, one will eventually, if not immediately, realize whether s/he is on the right path based on feedback from the real environment. Although the processing of external and self-realized feedback might be different, both of them may help navigators either confirm or recalibrate their navigation decisions derived from previous experience. To explore the function of feedback for navigation decision-making, the present study provided external feedback to half of the participants immediately after making a route decision at an intersection. Based on the immediate feedback, navigators might realize whether they have made a correct navigational decision. Feedback can help to judge the reliability of the navigator’s knowledge or the external guidance. However, participants without feedback would have few clues to do so, as routes in the present study expanded and eventually arrived at a certain destination in highly consistent environments (see Figures 1, 2). Thus the feedback condition represents the situation that navigators realized that they had made a wrong decision, while the no-feedback condition represents that they had no idea if they had made a correct decision. We would compare the two conditions to examine the effect of feedback on spatial decision-making with external guidance.

### The Effect of Direction Sources on Navigation Decision-Making

Previous findings suggest that the perceived credibility of an information source can influence human memory or decision-making (Carol et al., 2013; Blank et al., 2017). Studies on collaborative group work examined the effect of information source with a variety of types of information including word lists, pictures, videos, and spatial locations (Andersson and Rönnberg, 1995; Finlay et al., 2000; Shelton and McNamara, 2004; Wright and Klumpp, 2004; Thorley and Dewhurst, 2007; Ekeocha and Brennan, 2008; Galati et al., 2013; Sjolund et al., 2014). When recalling an event, others’ recollections may exert strong influence, even to the extent of discarding one’s own memory. Specifically, recalling a spatial array of objects after a collaborative discussion is usually better than when one reviews the information individually (Sjolund et al., 2014). The extent to which this happens can be affected by social factors, such as social status and relationships (Gabbert et al., 2007; Skagerberg and Wright, 2008; Wright et al., 2010; Carlucci et al., 2011; Wright, 2016; Hope and Gabbert, 2018). For example, intimate social partners, such as friends, spouses or siblings, influence one’s memory of an event to a greater extent than a stranger does (French et al., 2008; Hope et al., 2008; Peker and Tekcan, 2009).

Few studies examined whether or how spatial decision-making would be influenced by direction sources. Brunyé and his colleagues suggested that during wayfinding under uncertainty, navigators generally trust turn information provided by GPS and landmark information provided by humans (Brunyé et al., 2014). However, Brunyé et al.’s (2014) work used a route planning task, rather than a real wayfinding task, where participants had no navigation experience of the environments. The present study addressed this question by providing three types of direction sources, a GPS (Experiment 1), a stranger (Experiment 2), or a friend (Experiment 3) when participants were traveling through learned routes. These sources are typical directional guidance for wayfinding. GPS has become a primary external navigation guidance to human navigation. As GPS accuracy has largely improved, so has people’s reliance on it (Hoff and Bashir, 2015; Schaefer et al., 2016; Endsley, 2017). Studies exploring trust in automated systems suggest that people with low self-confidence trust automations to a greater extent than those with high self-confidence (de Vries et al., 2003; Hoff and Bashir, 2015). Obviously, problems arise when the route directions provided by GPS are incorrect. Research suggests that when automation systems make increasing mistakes, users would abandon the system and turn to their own knowledge (Lee and Moray, 1994; Brunyé et al., 2016; Chavaillaz et al., 2016).

Although studies show that automations were perceived as more reliable than humans (Lewandowsky et al., 2000; Madhavan and Wiegmann, 2007), very few studies examined the effect of direction sources on navigation decision-making from the perspective of social relations between navigators and their collaborators. Furthermore, researchers also pointed out that wayfinding as a social activity has been distinctly under-researched (Dalton et al., 2019). Most of existing research focuses on the comparison between navigating individually and collaborative navigation (Wunderlich and Reinelt, 1982; Reilly et al., 2009; Forlizzi et al., 2010; Haddington, 2012, 2013; He et al., 2015; Haghani and Sarvi, 2017). Their findings suggest that the presence, spatial abilities, and speech in communications of the collaborator could influence navigation strategies (Reilly et al., 2009; Forlizzi et al., 2010; Haddington, 2012). Therefore, the present study examined the social influence of direction sources on navigation decision-making and compared external guides of social significance with automated GPS.

### The Present Study

To summarize, the present study examined whether and how external direction sources influence navigation decisions while traveling in experienced environments. We hypothesized that the extent to which the external guidance affects navigation decision-making may be moderated by the availability of feedback and/or direction sources.

In addition, we examined the effects of route complexity by manipulating the number of intersections of a navigating route. Environmental complexity could influence wayfinding performance (Passini, 1980; Weisman, 1981; O’Neill, 1992; Carlson et al., 2010; Li and Klippel, 2016). Navigators in a complex, relative to a simpler, environment may have to make more cognitive efforts when integrating and applying spatial knowledge. Thus navigators may conform to external guides to reduce their cognitive cost when traveling through complex routes, especially when there was no immediate feedback provided.

We make the following hypotheses. First, navigators would show conformity to the external guidance by making more incorrect decisions with incorrect than with correct external guidance. Second, feedback on navigation decisions would provide opportunities for navigators to judge the reliability of both their own route knowledge and the external guidance, and recalibrate their navigation strategies. As such, conforming to external guidance should only be evident when feedback is not available. Third, the conformity to external guidance may vary as a function of direction sources (GPS, stranger, friend). As addressed earlier, navigators may have been used to follow GPS during navigation and considered it as a more reliable direction source than humans. Thus navigators may show high conformity to the external guidance when navigating with the GPS as in Experiment 1.

## Experiment 1: GPS as a Direction Source

### Method

#### Participants

Fifty-three undergraduates (21 males, 32 females) participated for monetary compensation. All participants provided informed consent before participating.

#### Materials

Road images were presented by E-prime 1.0 program from an overhead view and combined in succession to make up a specific route. Figure 1 shows possible road images, including straight routes and intersections. For straight routes, Figures 1A,B would be shown in succession to simulate a car moving forward. Changes in tree configuration between pictures could lead to the perception of a forward motion. The straight routes were always displayed vertically for all routes to keep an egocentric perspective for navigation. We had four types of intersection: three T-junctions and one four-way (see Figures 1C–F). As depicted, the type of T-junction dictated the turn options (left/right, right/straight, left/straight). For four-way intersections, the car could potentially turn left, right, or continue straight. The black arrows in the route images illustrated possible driving directions for each intersection, which were not displayed during formal navigation.

The road and intersection images composed six driving routes, three of which included 6 intersections and the other three included 10 intersections. There were six straight route images between two intersection images, and four straight route images connected to the last intersection at the end of a route. We presented three T-junctions and three four-way intersections in each 6-intersection route and six T-junctions and four four-way intersections in each 10-intersection route. The T-junctions were selected randomly from the above types and the presenting sequence of intersections was randomized across routes.

#### Design

The study used a 2 (route complexity: 6 vs. 10 intersections) × 2 (external guidance correctness: correct vs. incorrect) × 2 (turn feedback: feedback vs. no-feedback) mixed-design. The route complexity and guidance correctness served as within-participant variables and turn feedback served as a between-participant variable.

We manipulated the *route complexity* by having 6 or 10 intersections within a driving route. Participants needed to first learn the turn direction at each intersection within a route before proceeding to the navigation test. Thus, 10-intersection routes had a higher memory load than 6-intersection routes. The *external guidance correctness* was manipulated by presenting correct or incorrect direction by a simulated GPS in the testing phase (see Figure 2). The GPS would show correct directions at half of the intersections and incorrect directions at the other half. *Feedback* indicated whether participants had made correct turn decisions at intersections when navigating through learned routes. In the feedback condition, a turning image was shown after participants have turned at the intersection. The turning image would always indicate the correct turning direction, regardless of the participant’s decision. For instance, if the participant correctly turned right at an intersection, s/he would see the right turning image and then the straight route images to the next block (see Figure 2A). If a participant incorrectly turned left at an intersection, s/he would still see the right turning image showing the correct direction and then the route would continue straight to the next block (see Figure 2B). In seeing this, participants would realize that they had made an incorrect turn decision. In the no-feedback condition, no such turning images appeared. Participants would turn and then only see the straight route to the next block, but would not know if they turned to the correct direction (see Figure 2C).

Twenty-eight of 53 participants were assigned to the feedback group and the other 25 to the no-feedback group. In addition, to counterbalance the route complexity, 26 participants completed the 6-intersection routes first, while 27 participants completed the 10-intersection routes first.

#### Procedure and Coding

Participants completed the study in two alternating phases for each route: *learning phase* and *testing phase*. In the learning phase, participants received instructions on using arrow keys to move forward and/or turn at intersections. They traveled each route three times, following directions verbally given by an experimenter. Participants were instructed to remember each route during the learning phase. In Wang et al. (2018), over two thirds of participants could remember 16 locations after three rounds of navigating a virtual environment. Thus, traveling each route for three times in the present study may be enough for learning the 6- or 10-intersection routes. Then the experimenter would ask the participants to verbally report if they had learned the route. After the learning phase, participants were asked to repeat the route in the testing phase. An artificial GPS would periodically provide a correct or incorrect turn direction at each intersection. After participants completed the testing phase, they started learning a new route. They alternated between study and test until they finished all six routes. Their turning direction at each intersection was recorded to derive the accuracy of the navigation task. The response time (RT) at each intersection was recorded as the time lag between the presence of the intersection image and when the participant chose the turning direction.

### Results

After the learning phase, 50 and 43 of the 53 participants verbally confirmed that they had learned the 6- and 10-intersection routes, respectively. Analyses of the data of only those who confirmed their learning did not differ from analyses of all the participants. Thus, we use all the data in the current analyses. In addition, preliminary analyses based on the data of the 53 participants showed no effects of the display order of routes or gender, so we collapsed these factors in analyses. Analyses involved mixed model ANOVAs based on experiment design assessing accuracy and RT (see Table 1 for all effects). We only address significant effects here.

**TABLE 1:** Results of mixed ANOVA for Experiments 1–3.

	**Accuracy**			**RT**		
	***F***	***p***	***$\eta_{\text{p}}^{2}$***	***F***	***p***	***$\eta_{\text{p}}^{2}$***
**Experiment 1**						
Source (*df*)						
Correctness (1, 51)	18.790	< 0.001^∗∗^	0.269	3.950	0.052	0.072
Complexity (1, 51)	0.003	0.954	<0.001	0.072	0.790	0.001
Feedback (1, 51)	<0.001	0.999	<0.001	3.82	0.056	0.070
Correctness^∗^Feedback (1, 51)	17.839	< 0.001^∗∗^	0.259	5.884	0.019^∗^	0.103
Complexity^∗^Feedback (1, 51)	9.674	0.003^∗∗^	0.159	1.680	0.201	0.032
Correctness^∗^Complexity (1, 51)	4.858	0.032^∗^	0.087	0.302	0.585	0.006
Correctness^∗^Complexity^∗^Feedback (1, 51)	1.517	0.224	0.029	0.223	0.639	0.004
**Experiment 2**						
Source (*df*)						
Correctness (2, 88)	8.647	< 0.001^∗∗^	0.164	0.316	0.730	0.007
Complexity (1, 44)	0.279	0.600	0.006	0.140	0.710	0.003
Feedback (1, 44)	0.150	0.701	0.150	19.68	< 0.001^∗∗^	0.309
Correctness^∗^Feedback (2, 88)	8.397	< 0.001^∗∗^	0.160	0.786	0.459	0.018
Complexity^∗^Feedback (1, 44)	3.871	0.055	0.081	4.326	0.043^∗^	0.090
Correctness^∗^Complexity (2, 88)	4.559	0.013^∗^	0.094	0.843	0.434	0.019
Correctness^∗^Complexity^∗^Feedback (2, 88)	0.082	0.922	0.002	0.830	0.440	0.019
**Experiment 3**						
Source (*df*)						
Correctness (2, 98)	3.566	0.032^∗^	0.068	2.180	0.119	0.043
Complexity (1, 49)	3.988	0.051	0.075	4.357	0.042^∗^	0.082
Feedback (1, 49)	6.169	0.016^∗^	0.112	6.566	0.014^∗^	0.118
Correctness^∗^Feedback (2, 98)	7.680	0.001^∗∗^	0.135	0.465	0.629	0.009
Complexity^∗^Feedback (1, 49)	0.308	0.582	0.006	0.677	0.415	0.014
Correctness^∗^Complexity (2, 98)	0.162	0.851	0.003	0.665	0.517	0.013
Correctness^∗^Complexity^∗^Feedback (2, 98)	0.072	0.931	0.001	3.361	0.039	0.064

*The table shows the results of repeated measures ANOVA for Experiments 1–3. Correctness is for guidance correctness, complexity is for route complexity, feedback is for turn feedback. ^∗^Significant at α < 0.05; ^∗∗^Significant at α < 0.01.*

**FIGURE 3:**
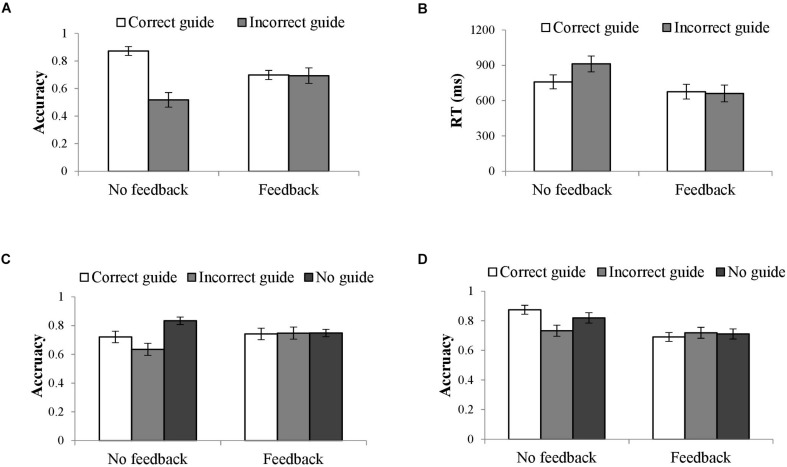
Interaction between guidance correctness and turn feedback in accuracy for Experiments 1, 2, and 3 **(A,C,D)** and in RT for Experiment 1 **(B)**.

Analyses showed an effect of external guidance correctness on turn decision accuracy. Participants showed higher accuracy when the GPS provided correct directions (*M* = 0.79, 95% confidence interval (CI) = [0.74, 0.83]) than incorrect directions (*M* = 0.61, 95%CI = [0.53, 0.68]). The effect of guidance correctness was qualified by an interaction between correctness and feedback for both accuracy and RT. As Figures 3A,B illustrates, participants in the no-feedback condition performed better with correct (*M* = 0.87, 95%CI = [0.81, 0.94]) than with incorrect guidance (*M* = 0.52, 95%CI = [0.41, 0.63], *p* < 0.001). RT mirrored this effect, showing faster RT with the correct (*M* = 760.3 ms, 95%CI = [641.0, 877.6]) compared to incorrect guidance (*M* = 911.8 ms, 95%CI = [776.5, 1047.2]), *p* = 0.002. With feedback, guidance correctness did not affect performance in both accuracy and RT, *p*s > 0.5.

**FIGURE 4:**
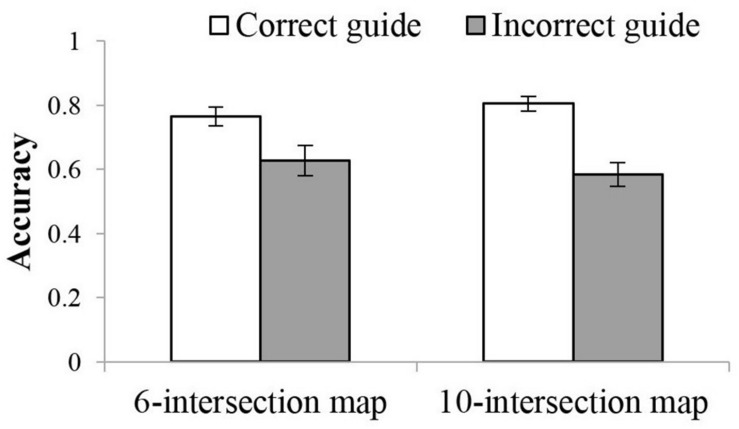
Interaction between route complexity and guidance correctness in accuracy in Experiment 1.

Guidance correctness also interacted with route complexity in accuracy. Although correctness affected accuracy regardless of complexity for 10- and 6-intersection routes, *p* < 0.001 and *p* < 0.01 respectively, the difference between the correct and incorrect guidance was greater for 10-intersection (*M*_correct_ = 0.81, 95%CI = [0.76, 0.85]; *M*_incorrect_ = 0.58, 95%CI = [0.51, 0.66]) routes than that for 6-intersection routes (*M*_correct_ = 0.77, 95%CI = [0.70, 0.83], *M*_incorrect_ = 0.63, 95%CI = [0.53, 0.72], see Figure 4).

**FIGURE 5:**
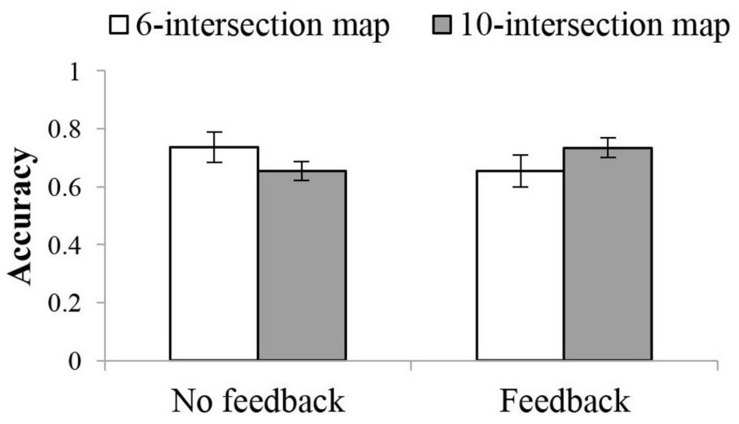
Interaction between route complexity and feedback in accuracy in Experiment 1.

Accuracy also showed an interaction between feedback and route complexity. Participants in the no-feedback condition had a higher accuracy with the 6-intersection (*M* = 0.74, 95%CI = [0.65, 0.82]) than the 10-intersection routes (*M* = 0.65, 95%CI = [0.59, 0.72]), *p* = 0.025. By contrast, with feedback, they performed better with the 10-intersection (*M* = 0.74, 95%CI = [0.67, 0.80]) than the 6-intersection routes (*M* = 0.66, 95%CI = [0.56, 0.75]), *p* = 0.041, see Figure 5).

### Discussion

The results of Experiment 1 showed that without feedback during the testing phase, the accuracy of turning decisions changed as a function of the guidance correctness, supporting navigators’ reliance on the GPS. If the participant exclusively followed the incorrect guidance, then the accuracy would be zero. That is, the lower the accuracy is, the more likely participants may follow the external guidance. For the incorrect guidance condition, participants without feedback had a much lower accuracy (52%) than those who had feedback for navigation decisions (69%). Experiment 1 suggests that, despite claiming to have learned a route, participants were more likely to follow the GPS to complete navigation than to rely on their own spatial knowledge.

Although navigators appear to rely on the GPS, route knowledge was evident. Navigators took more time to decide on a turning direction when the GPS gave incorrect directions than when it gave correct directions. Such a lag in RT suggests that participants may realize the conflict between the external guidance and their route knowledge. Notably, feedback mitigated the impact of the external guidance. This may have occurred because participants took a navigation strategy based on the evaluation of the external guidance and their own route knowledge through the feedback.

## Experiment 2: Stranger as a Direction Source

Global Positioning System devices have become highly reliable navigation guidance and people often believe that GPS devices always show correct directions (e.g., Hoff and Bashir, 2015). As such, most navigators trust GPS as a direction source. This may especially be the case for those who have low confidence in their sense of direction or show a lack of knowledge about spatial environments (de Vries et al., 2003; Körber et al., 2018). Accordingly, navigators may consider GPS to be much more reliable and trustworthy than their own capability of wayfinding (Lewandowsky et al., 2000; de Vries et al., 2003; Madhavan and Wiegmann, 2007). If a GPS is not available, people may turn to another person for route directions (Brunyé et al., 2014). However, the extent to which one weighs someone else’s navigation directions over his/her own spatial knowledge is not clear. Experiment 2 examined how external direction guidance provided by a stranger affects navigation decision-making. Studies on memory conformity suggest that messages conveyed by strangers in collaborative tasks can distort memory, regardless of the reliability of the messages (French et al., 2008; Hope et al., 2008; Peker and Tekcan, 2009; Jaeger et al., 2012; Kieckhaefer and Wright, 2014; Numbers et al., 2014). Thus, a stranger, presumably having experience with the route, provided navigational directions in Experiment 2. If the participant trusts the stranger more than their own route knowledge, then their performance would match Experiment 1. This would especially be the case without feedback during navigation.

### Method

#### Participants

Fifty-two new undergraduates (31 males, 21 females), who did not take part in Experiment 1, participated individually for monetary compensation. All participants read and signed informed consent before the experiment.

#### Stimulus and Design

The routes matched those used in Experiment 1. The stranger’s navigation directions consisted of verbal recordings, done by one experimenter, stating “turn left,” “turn right,” or “keep going forward,” presented through earphones.

The study design matched Experiment 1, with one exception. We added one level to external guidance correctness by providing no guidance at some intersections. There were roughly equal numbers of correct and incorrect guidance and equal numbers of guidance and no guidance. More specifically, across the three 6-intersection routes, 5 intersections gave correct directions, 4 gave incorrect directions, and 9 had no guidance. Across the three 10-intersection routes, 7 intersections gave correct directions, 8 gave incorrect directions, and 15 had no guidance. Thus, the study used a 2 (route complexity: 6 vs. 10 intersections) × 3 (guidance correctness: correct vs. incorrect vs. no guidance) × 2 (turn feedback: feedback vs. no-feedback) mixed-design. As in Experiment 1, route complexity and guidance correctness served as within-participant variables and turn feedback served as a between-participant variable. Also, participants in the feedback group would see turning images after making route decisions as in Experiment 1, while those in the no-feedback group would not see the turning images.

Half of the 52 participants were assigned to the feedback group and the other half to the no-feedback group. Furthermore, the order of the two route complexity conditions (i.e., 6-intersection vs. 10-intersection) was counterbalanced across participants within the feedback/no-feedback groups. Twenty-six participants completed the 6-intersection routes first and 26 completed the 10-intersection routes first.

#### Procedure

Participants completed the study in two phases: learning and testing. The learning phase was identical to those in Experiment 1. Before the testing phase, we told participants that one participant, who had finished the study already, left some route directions. They would hear these directions through earphones while driving. Then participants were asked to repeat the route. We neither implied that the verbal guidance was correct nor asked participants to follow the guidance. After finishing one learned route, participants proceeded to the next new route until they finished all six routes.

### Results

The data of six participants (five males, one female) were eliminated from the following analysis, as their RTs were greater than the group average plus three SDs. Forty-four and 40 of the remaining 46 participants verbally confirmed that they had learned the 6- and 10-intersection routes. Results of only those who confirmed their learning did not differ from the results of all the participants. Thus, we again use all the data for analyses. Preliminary analysis of the 46 participants showed no effects of task order or gender. Therefore, analyses collapsed across these variables. Analyses consisted of mixed model ANOVAs assessing accuracy and RT based on the experiment design (see Table 1 for all effects). We again only address significant effects here.

The analyses showed an effect of guidance correctness in accuracy. Pairwise comparisons showed the highest accuracy with no guidance (*M* = 0.79, 95%CI = [0.75, 0.83]) compared to either a correct or incorrect guidance, *p* = 0.019 and *p* = 0.001. The correct guidance led to higher accuracy (*M* = 0.73, 95%CI = [0.68, 0.79]) than the incorrect guidance (*M* = 0.69, 95%CI = [0.63, 0.75]), *p* = 0.057. As in Experiment 1, this effect was qualified by the interaction between guidance correctness and feedback. As Figure 3C illustrates, without feedback, participants showed the highest accuracy without external guidance (*M* = 0.83, 95%CI = [0.78, 0.89]), compared to correct or incorrect guidance condition, *p* = 0.007 and *p* < 0.001. With correct guidance (*M* = 0.72, 95%CI = [0.64, 0.80]) participants had higher accuracy than with incorrect guidance (*M* = 0.64, 95%CI = [0.55, 0.72]), *p* = 0.014. With feedback, no significant differences emerged, *p*s > 0.5.

Guidance correctness also interacted with route complexity for accuracy. For the 6-intersection routes, participants had higher accuracy with no guidance (*M* = 0.80, 95%CI = [0.76, 0.84]) than with correct (*M* = 0.71, 95%CI = [0.65, 0.78]), *p* = 0.009 or incorrect guidance (*M* = 0.72, 95%CI = [0.65, 0.80]), *p* = 0.094, although the latter one was not statistically significant. The accuracy of correct and incorrect guidance conditions did not differ, *p* > 0.5. With the 10-intersection routes, the incorrect guidance condition (*M* = 0.66, 95%CI = [0.60, 0.73]) led to the lowest accuracy, compared to having a correct (*M* = 0.75, 95%CI = [0.69, 0.81]), *p* < 0.001 or no guidance (*M* = 0.79, 95%CI = [0.75, 0.83]), *p* < 0.001. The latter two conditions did not differ, *p* > 0.5.

The analysis also showed an interaction between turn feedback and route complexity, although the results of simple effect analyses were not statistically significant for both feedback and no-feedback condition. Without feedback, participants took longer to respond with the 10-intersection (*M* = 839.8 ms, 95%CI = [753.2, 926.5]) than the 6-intersection routes (*M* = 790.5 ms, 95%CI = [721.1, 860.0]), *p* = 0.234. For the feedback condition, participants took longer with 6-intersection (*M* = 604.6 ms, 95%CI = [535.2, 674.1]) than with 10-intersection routes (*M* = 570.4, 95%CI = [483.7, 657.0]), *p* = 0.090.

Turn feedback affected RT. Participants responded much faster with feedback (*M* = 587.5 ms, 95%CI = [514.4, 660.6]) than without feedback (*M* = 815.2 ms, 95%CI = [742.0, 888.3]). Experiment 1 saw a similar trend, although it was not statistically significant (*p* = 0.056). Participants responded faster with feedback (*M* = 668.5 ms, 95%CI = [543.8, 793.2]) than without feedback (*M* = 835.6 ms, 95%CI = [717.7, 953.4]) in Experiment 1.

### Discussion

Experiment 2 results, similar to Experiment 1, suggest that without feedback, participants were affected by the correctness of external direction guidance, and in this case, a stranger’s directions to navigate. Further, the no-guidance condition reflected participants’ route knowledge. They performed best without the stranger’s guidance, even compared to the conditions with correct guidance. Participants can simply use their own route knowledge without the interference from external guidance. This supports their claims of having learned the route. Interestingly, this finding implies that when the external guidance is available, navigators have difficulty in resisting following its direction, regardless of the correctness. In other words, they conform to the external source consistently, even when their route knowledge is good. Another factor to consider is that incorrect and correct guidance are intermixed within a route. If the participants completely trusted and followed the correct guidance, then they would have the highest accuracy. However, the results did not support this assumption. As the incorrect and correct guidance were presented in a within-participant design, we inferred that the incorrect guidance might influence the reliability of external guidance to navigators. Thus navigators did not follow the correct guidance readily. The correct guidance, which was not consistent with participant’s knowledge, may interfere with their decision-making. But they cannot completely ignore the external guidance, especially without feedback. Thus the struggling between the navigational guidance and navigator’s own knowledge would decrease the accuracy of the correct guidance condition.

However, with feedback, participants may evaluate the reliability of the direction source by potentially matching it to their route knowledge. Once they realize that the direction source could be wrong, they might have ignored it entirely and relied on their own decisions. Thus, such post-decision feedback could potentially improve a navigator’s confidence in their route knowledge, especially after making a correct decision. Supporting this, accuracy did not differ across the three guidance correctness conditions when feedback was present.

## Experiment 3: Friend as a Direction Source

As mentioned earlier, social relations between two people can affect conformity behaviors and task accuracy (Finlay et al., 2000; Wright and Klumpp, 2004; Thorley and Dewhurst, 2007; Ekeocha and Brennan, 2008; French et al., 2008; Hope et al., 2008; Peker and Tekcan, 2009; Kieckhaefer and Wright, 2014; Sjolund et al., 2014). When people discussed an event with a friend, versus a stranger, they were more likely to trust the friend’s information, even when it contained inaccurate information. People considered the acquaintance’s knowledge to be more accurate and reliable than their own and that the acquaintance was more trustworthy than a stranger (French et al., 2008; Hope et al., 2008). Kieckhaefer and Wright (2014) suggested that when there was a positive, rather than negative, relationship between participants and collaborators, participants showed greater eyewitness accuracy and were less suggestible. Further studies have also found that with a likable partner, participants may feel more relaxed and less anxious, allowing them to concentrate more on the specific task of recalling relevant knowledge (Collins et al., 2002; Vallano and Schreiber Compo, 2011; Kieckhaefer et al., 2013). Thus, in the present experiment, we asked volunteers to make verbal recordings of the route and then to invite close friends to take part in the study. The positive relationship between the volunteers as a direction source and their friends as navigators may make the experiment situation more comfortable and enhance navigation decisions. We predicted that participants would still follow the external guidance, even when it was incorrect, with a friend as a direction source.

### Method

#### Participants

Fifty-two new undergraduates (25 males, 27 females), who did not take part in Experiments 1 and 2, participated individually for monetary compensation. All participants completed an informed consent prior to the experiment.

#### Stimulus and Design

The routes matched those used in Experiments 1 and 2. We asked five participants, who had not learned the routes, to record verbal guidance of directions, including correct or incorrect directions. Thus the correctness of verbal guidance matched those in Experiment 2. They then invited their friends to take part in the experiment without informing them the procedure of the study. All other stimuli and the experiment design matched those of Experiment 2.

#### Procedure

The learning phase matched Experiments 1 and 2. Before the testing phase, we told the participant that his/her friend had finished the study and left some route directions by verbal recording. Participants would hear these directions through earphones during the testing phase. Then participants were asked to repeat the driving route. After participants repeated the route, they would start a new one until they finished all the six routes.

### Results

The data of one female participant was eliminated from the following analysis, because her RTs were greater than the mean plus three SDs. After the learning phase, 49 and 43 of the left 51 participants verbally confirmed that they had learned the 6- and 10-intersection routes, respectively. Analyses of the data of only those who confirmed their learning did not differ from the results of all participants. Thus, we use all data in the analyses. Preliminary analysis of the 51 participants showed no effect of task order or gender, so analyses collapsed across these variables. Analyses consisted of mixed model ANOVAs on accuracy and RT based on the study design (see Table 1 for all effects).

The analyses showed an effect of guidance correctness in accuracy. Pairwise comparisons showed the lowest accuracy with the incorrect guidance (*M* = 0.73, 95%CI = [0.67, 0.78]), compared to the correct (*M* = 0.78, 95%CI = [0.74, 0.83]), *p* = 0.021, or no guidance (*M* = 0.77, 95%CI = [0.72, 0.82]), *p* = 0.083, although the latter one was not statistically significant. The accuracy of correct and no guidance conditions did not differ from one another, *p* > 0.1. As in Experiments 1 and 2, this effect was qualified by an interaction between guidance correctness and turn feedback. Without feedback, accuracy based on guidance correctness differed, but with feedback it did not, *p*s > 0.5. As Figure 3D illustrates, participants without feedback had the highest accuracy with the correct guidance (*M* = 0.88, 95%CI = [0.81, 0.94]) compared to the incorrect guidance (*M* = 0.73, 95%CI = [0.66, 0.81]), *p* < 0.001, and to the no guidance condition (*M* = 0.82, 95%CI = [0.75, 0.89]), *p* = 0.156, although the difference was not statistically significant. Further, they were more accurate with no guidance compared to the incorrect guidance, *p* = 0.024.

A main effect of feedback was observed on accuracy. Participants with feedback were less accurate (*M* = 0.71, 95%CI = [0.65, 0.77]) and had shorter RT (*M* = 804.5 ms, 95%CI = [668.6, 940.3]) than those without feedback (*M* = 0.81, 95%CI = [0.75, 0.87]; *M* = 1051.9 ms, 95%CI = [913.3, 1190.5]). This may show a speed-accuracy tradeoff for the decision-making process.

### Discussion

Experiment 3 again showed reliance on external guidance when navigating through the learned routes. Participants’ navigation decisions were more influenced by their friend’s direction guidance without feedback, compared to feedback conditions.

Further, knowing the direction source, compared to a stranger or a GPS, impacted use of the guidance. Without feedback, Experiment 3 found no accuracy difference between the correct and no guidance condition. This is unlike Experiment 2, wherein the no guidance condition had much higher accuracy than the correct guidance condition. Compared to strangers, knowing the direction source may instill more trust in the guidance.

## Comparison Across Studies: The Effect of Information Sources

To investigate the effect of direction sources, we conducted a comparison across Experiments 1 to 3, using the direction source as a between-participant variable. As Experiment 1 did not include the no-guidance condition, we removed the no-guidance condition from Experiments 2 and 3 for this analysis. Thus, the analyses consisted of 2 (route complexity: 6 vs. 10 intersections) × 2 (guidance correctness: correct vs. incorrect guidance) × 2 (turn feedback: feedback vs. no-feedback) × 3 (direction source: GPS vs. stranger vs. friend) mixed model ANOVAs on accuracy and RT. All of the effects are shown in Table 2.

**TABLE 2:** Results of mixed ANOVA for the across-study analysis.

	**Accuracy**			**RT**		
**Across-Study Analysis**	***F***	***p***	***$\eta_{\text{p}}^{2}$***	***F***	***p***	***$\eta_{\text{p}}^{2}$***
Source (*df*)						
Correctness (1, 143)	26.321	< 0.001^∗∗^	0.155	4.203	0.042^∗^	0.029
Complexity (1, 143)	0.983	0.323	0.007	1.567	0.213	0.011
Feedback (1, 143)	0.221	0.639	0.002	18.497	< 0.001^∗∗^	0.115
Source (2, 143)	1.718	0.183	0.023	7.093	0.001^∗∗^	0.090
Feedback^∗^Source (2, 143)	2.684	0.072	0.036	0.268	0.765	0.004
Correctness^∗^Source (2, 143)	6.094	0.003^∗∗^	0.079	2.350	0.099	0.032
Correctness^∗^Feedback (1, 143)	32.200	< 0.001^∗∗^	0.184	1.278	0.260	0.009
Complexity^∗^Source (2, 143)	0.565	0.570	0.008	1.522	0.222	0.021
Complexity^∗^Feedback (1, 143)	7.695	0.006^∗∗^	0.051	0.032	0.859	<0.001
Complexity^∗^Correctness (1, 143)	7.000	0.009^∗∗^	0.047	0.009	0.926	<0.001
Correctness^∗^Source^∗^Feedback (2, 143)	4.551	0.012^∗^	0.060	2.006	0.138	0.027
Complexity^∗^Source^∗^Feedback (2, 143)	2.177	0.117	0.030	1.303	0.275	0.018
Complexity^∗^Correctness^∗^Source (2, 143)	1.653	0.195	0.023	0.407	0.666	0.006
Complexity^∗^Correctness^∗^Feedback (1, 143)	0.177	0.675	0.001	4.663	0.032^∗^	0.032
Complexity^∗^Correctness^∗^Source^∗^Feedback (2, 143)	0.710	0.493	0.010	2.588	0.079	0.035

*The table shows the results of repeated measures ANOVA for the across-study analysis. Validity is for guidance correctness, complexity is for route complexity, feedback is for turn feedback, and source is for information sources. ^∗^Significant at α < 0.05; ^∗∗^Significant at α < 0.01.*

Consistently, the analyses showed an effect of guidance correctness in both accuracy and RT. Participants showed higher accuracy when the external guidance provided correct (*M* = 0.77, 95%CI = [0.74, 0.79]) than incorrect directions (*M* = 0.67, 95%CI = [0.64, 0.71]). They also took less time to make decisions with correct (*M* = 765.1 ms, 95%CI = [718.5, 811.7]) compared to incorrect guidance (*M* = 809.3 ms, 95%CI = [754.7, 864.0]). Again, this effect was qualified by an interaction between guidance correctness and turn feedback in accuracy. Without feedback, participants made more accurate decisions with correct (*M* = 0.82, 95%CI = [0.78, 0.86]) than with incorrect guidance (*M* = 0.63, 95%CI = [0.58, 0.68]), *p* < 0.001. With feedback, no difference was observed, *p*s > 0.5.

**FIGURE 6:**
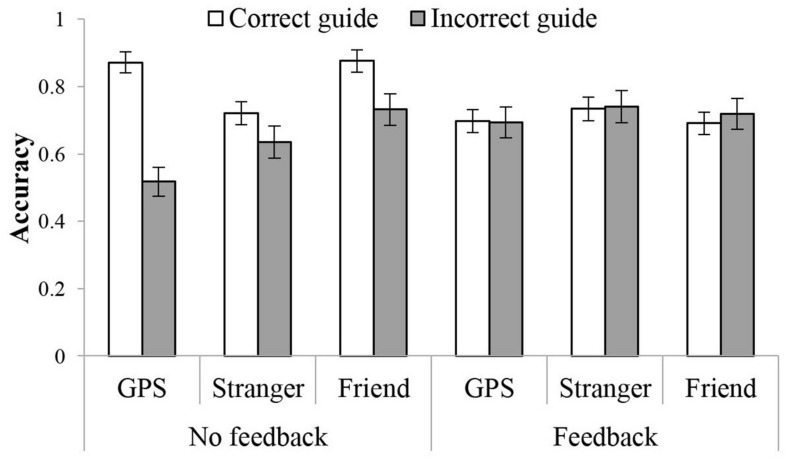
Three-way interaction between guidance correctness, turn feedback, and information sources in accuracy in the across-study analysis.

More importantly, the analyses showed an interaction in accuracy between the guidance correctness and direction source. This interaction was qualified by a three-way interaction between guidance correctness, direction source, and turn feedback. Simple effect analyses suggest an interaction between guidance correctness and direction source when there was no feedback, *F*(2,72) = 7.67, *p* = 0.001, $\eta_{\text{p}}^{2}$ = 0.18, but not when there was feedback, *F*(2,71) = 0.26, *p* = 0.780, $\eta_{\text{p}}^{2}$ = 0.01. As Figure 6 illustrates, participants with feedback had roughly equivalent accuracy for correct and incorrect guidance, *p*s > 0.5 (see Table 3 for means related to this interaction). Without feedback, participants had much higher accuracy with correct than incorrect guidance, particularly with the GPS or Friend as the direction source, *p* < 0.001 and *p* = 0.009, respectively. The difference was a little weak with a Stranger as the direction source, *p* = 0.119. Furthermore, without feedback and having correct guidance, participants had higher accuracy with GPS or Friend than with Stranger, *p* = 0.004 and *p* = 0.005, respectively. When the external guidance was incorrect, people had lower accuracy with the GPS than with Friend, *p* = 0.022, neither of which differed from the Stranger condition, *p*s > 0.5.

**TABLE 3:** Mean accuracy for Correct and Incorrect guidance conditions with or without feedback.

	**No feedback**		**Feedback**	
	**Correct**	**Incorrect**	**Correct**	**Incorrect**
**Sources**	**guidance**	**guidance**	**guidance**	**guidance**
GPS (*M*)	0.87	0.52	0.70	0.69
95%CI	[0.81, 0.94]	[0.43, 0.60]	[0.63, 0.76]	[0.60, 0.78]
Stranger (*M*)	0.72	0.64	0.73	0.74
95%CI	[0.65, 0.79]	[0.54, 0.73]	[0.67, 0.80]	[0.65, 0.84]
Friend (*M*)	0.88	0.73	0.69	0.72
95%CI	[0.81, 0.94]	[0.64, 0.83]	[0.63, 0.76]	[0.63, 0.81]

As in Experiments 1 and 2, the cross-study analysis showed an interaction between route complexity and feedback. Without turn feedback, participants showed lower accuracy with 10- (*M* = 0.70, 95%CI = [0.66, 0.74]) than with 6-intersection routes (*M* = 0.76, 95%CI = [0.71, 0.80]), *p* = 0.008. With feedback, this difference was not significant (*M*_10__–intersection_ = 0.73, 95%CI = [0.69, 0.76], *M*_6__–intersection_ = 0.70, 95%CI = [0.65, 0.74]), *p* = 0.224. Route complexity also interacted with guidance correctness. The difference between correct and incorrect guidance was larger for 10- (*M*_correct_ = 0.77, 95%CI = [0.74, 0.80], *M*_incorrect_ = 0.65, 95%CI = [0.61, 0.69]), *p* < 0.001, than for 6-intersection routes (*M*_correct_ = 0.76, 95%CI = [0.72, 0.79], *M*_incorrect_ = 0.70, 95%CI = [0.65, 0.74]), *p* = 0.008.

The cross-study analysis also revealed that the direction source impacted RT. Participants took much more time to make decisions with Friend (*M* = 908.7 ms, 95%CI = [829.2, 988.1]), compared to GPS (*M* = 752.0 ms, 95%CI = [674.8, 829.3]), *p* = 0.006, and Stranger (*M* = 701.0 ms, 95%CI = [618.3, 783.7]), *p* < 0.001, respectively, which did not differ from each other, *p* = 0.374. As in the separate analyses, participants responded faster with feedback (*M* = 687.0 ms, 95%CI = [621.6, 752.3]) than without feedback (*M* = 887.5 ms, 95%CI = [822.5, 952.5]).

### Discussion

The cross-study comparisons supported the interaction between feedback and guidance correctness, showing that navigators rely on external guidance when feedback is not available. These results confirmed findings of the individual studies and further showed how direction sources can influence navigation decision-making.

New with this analysis, the extent of dependency on external guidance might be moderated by the direction sources. Under incorrect guidance condition, participants showed highest accuracy with their friends as a direction source, compared to the GPS and Strangers. Being familiar with the direction source may make the situation less stressful, allowing people to focus more on the task (Vallano and Schreiber Compo, 2011; Kieckhaefer et al., 2013). In contrast, participants seemed to rely on the GPS without considering its correctness. There are even stories of people driving into a lake because their GPS system “told them to” (Kircher, 2018). Here, people made less accurate route decisions when the GPS provided incorrect directions. The reduced accuracy with GPS implies that navigators are over-dependent on and/or over-confident in GPS, allocating few cognitive resources into processing navigation-related spatial information themselves (Hoff and Bashir, 2015; Schaefer et al., 2016). Increased reliance on GPS guidance may not only result in negative outcomes for navigation, but also interfere with the integration of spatial knowledge (e.g., Gardony et al., 2013, 2015). Finally, participants may have trusted and used directions from GPS and friends, to a larger degree than those from a stranger. The uncertainty on strangers likely results in hesitations in using their provided directions during navigation, even when the directions are correct (Kieckhaefer and Wright, 2014; Zawadzka et al., 2016).

## General Discussion

### External Guidance Influences Navigation Decisions on Experienced Routes

Three studies explored whether and how people would use external direction guidance provided by various sources. Participants navigated through a simulated route in the learning phase and then repeated the route accompanied by correct or incorrect external guidance. If the navigators’ route knowledge is susceptible to be interfered, as seen in other fields, they would take the direction provided by external guidance. Our results consistently suggest that people have a disposition to use external guidance, especially when there was no immediate feedback for navigation decisions. Although most participants verbally indicated that they had learned the route, they might have less confidence in their own route knowledge than in the external navigation guidance. Nevertheless, RT results suggest that participants sometimes recognized that the external guidance conflicted with their knowledge, as they took more time to make a decision with incorrect guidance, especially when feedback was absent.

The immediate feedback after navigation decisions played a critical role in whether participants depended on the external guidance or not. As addressed above, the navigation performance without feedback changed as a function of the guidance correctness, indicating that participants relied more on external guidance than their own knowledge. Without feedback, navigators cannot judge the accuracy of either one’s own route knowledge or the external guidance. However, with feedback, participants could use the feedback as objective criteria to judge the correctness of their route knowledge and the guidance. Additionally, even in the absence of explicit feedback, retrieval (deciding a turn) can be benefit learning in that error-correction learning provides some opportunities for learning (e.g., Carrier and Pashler, 1992). Such processing of retrieval practice occurred repeatedly at intersections during navigation, facilitating to develop a solid navigation strategy and decreasing reliance on the external guidance. This was evident in the similar navigation performance when the direction giver provided correct versus incorrect direction. As seen in research on the *testing effect*, positive feedback can help improve the memory of studied and correct information. In our case, the positive feedback may then strengthen the route knowledge after navigation (Carrier and Pashler, 1992; Chan et al., 2006; Karpicke and Roediger, 2008; Chan, 2009; Thomas et al., 2010; Kelly et al., 2015). Thus, having feedback or not may change people’s navigation strategies. People appear to default to the external guidance, likely because it is cognitively easier.

### The Effect of Direction Source on Navigation Decision-Making

As addressed earlier, people are affected by information sources when attempting to collaboratively recall a specific event (Gabbert et al., 2007; French et al., 2008; Hope et al., 2008; Skagerberg and Wright, 2008; Peker and Tekcan, 2009; Carlucci et al., 2011; Carol et al., 2013; Kieckhaefer et al., 2013; Kieckhaefer and Wright, 2014; Wright, 2016; Blank et al., 2017). The present study expands research on information sources to navigation decision-making. Research on automation dependency suggests that people tend to depend on automation when completing work that involves technology. This is especially true for those with low confidence in their own ability and/or with less knowledge of the relative fields (Hoff and Bashir, 2015; Schaefer et al., 2016; Endsley, 2017). The current research also showed that participants without feedback consistently took directions provided by the external guidance. Anecdotally, many participants expressed low confidence in their spatial knowledge and abilities when instructed to learn the routes. Interestingly, Experiments 2 and 3 showed decent performance when people had to use their own knowledge. Specifically, participants performed as well as or even better without external guidance than with correct guidance. This may imply that their route knowledge was more reliable than they estimated. Underestimating one’s spatial knowledge might contribute to over-reliance on external aids like GPS systems during navigation. Over-reliance on GPS also hinders spatial knowledge development (e.g., Gardony et al., 2013, 2015). According to this finding, once one has learned the route, it is better off not giving them any external guidance than having a guidance at all, whether the guidance is valid or not.

The cross-study comparison suggests that the direction source affects people’s reliance on the external guidance. Participants took more time making decisions with a friend as the direction source, compared to a GPS or a stranger. People may make more cognitive efforts during navigation with a friend, anticipating future interactions (Collins et al., 2002; Vallano and Schreiber Compo, 2011; Kieckhaefer et al., 2013; Kieckhaefer and Wright, 2014). This was evident in the interaction between direction source, guidance correctness and feedback. Further, based on past experience, people can better assess the reliability of directions provided by a friend compared to those provided by a stranger, considering things like whether the friend has a good sense of direction or spatial ability. This experience with friends may then affect how the navigator responded when the direction provided by the friend was either consistent or inconsistent with their own route knowledge. Further studies could explore this issue by manipulating the sense of direction of the friend who provides directions.

Compared with GPS and friend, participants trusted a stranger’s guidance the least, as they showed the lowest accuracy with correct guidance provided by strangers. Even though people still used incorrect guidance from a stranger, correct guidance from the stranger did not improve performance as much as the same information from a GPS or friend. Participants might not have blindly trusted all the guidance provided by the stranger, because otherwise they would have done best with the correct guidance. This implies that participants may make the navigational decisions with the interference of external guidance provided by a stranger, leading to a decreasing accuracy of navigation decision-making.

### The Effect of Route Complexity on Navigation Decision-Making

An environment’s complexity affects wayfinding performance (O’Neill, 1992; Li and Klippel, 2016; Slone et al., 2016). In the present study, the implication of environment complexity is twofold. First, route complexity increased the performance discrepancy between the correct and incorrect guidance. The effect of guidance correctness was more salient when navigating 10-intersection routes, indicating that participants relied more on the external guidance in complicated environments. More complex environments may require more cognitive resources to learn. Participants then have more difficulties recalling all the information and/or may have less confidence in their knowledge. In this case, the external guidance would decrease cognitive loads, resulting in following the guidance regardless of its correctness. Thus, the greater likelihood of following external guidance in a more complex environment may lead to greater influence on spatial decision-making. The present study suggests that information complexity would increase the likelihood of interference in recalling relevant knowledge.

Second, the route complexity interacted with feedback in recalling route directions. One interesting result of the present study is that with feedback, participants showed higher accuracy with 10- than with 6-intersection routes, the opposite of the results without feedback. As addressed earlier, immediate feedback at each intersection helps navigators assess and potentially correct their knowledge. Therefore, larger environments provide more opportunities to calibrate navigate strategies, potentially leading to better performance than that in simple environments.

### Limitations

One limitation for the present study is that individual differences in the sense of direction likely impact the extent to which people use external guidance. Navigators with a good sense of direction may have higher confidence in their spatial memory and are less influenced by external guidance. Further research should explore how individual differences affect responses to external guidance.

The present research explores only the first step in how external guidance may affect navigation decisions. This work examined whether and how people used external directions when navigating through learned routes. The present study however did not include a memory test, which may provide opportunities for participants to evaluate their route knowledge. We examined the state that participants considered that they had learned the routes while they may still be uncertain or have low confidence of their spatial knowledge. Participants confirmed that they had learned the routes with verbal reports, which also suggests that subjective evaluation of one’s spatial knowledge may not be accurate. If participants consciously knew that they had not learned the routes very well through memory tests, then they might have a high disposition to conform to external guidance before the test navigation. Thus we did not have a memory test after learning or testing phase. Additionally, assessing the same route twice, once to see whether they have remembered it, and once to present the external guidance may make our experimental manipulation (i.e., external guidance) void. While most participants reported having learned the routes, future research could use a criterial learning task, like a map drawing task or map reconstruction task to assess the extent to which people had accurate route knowledge. These manipulations can take this present work one step further to explore whether using incorrect external guidance would distort spatial knowledge.

### Summary

The present study investigates the effect of feedback and direction sources on the usage of external guidance during navigating through learned routes. Results of three experiments suggest that people without immediate navigation feedback tend to rely on external navigation guidance. This work also shows that people use feedback to evaluate the reliability of their own route knowledge and the external guidance to develop an efficient navigation strategy. Finally, not all external guidance is treated equally. Trust in external guidance varies as a function of the perceived reliability of direction sources and their relationships with the navigator. Navigators trust GPS most, but perform best with a friend as the direction source. The present study extends the research of navigation as a social activity by examining the authority and social significance of direction sources.

## Data Availability

The raw data supporting the conclusions of this manuscript will be made available by the authors, without undue reservation, to any qualified researcher.

## Ethics Statement

This study was carried out in accordance with the recommendations of Guidelines for Human Subject Research, Institutional Review Board of Department of Psychology, Sun Yat-sen University with written informed consent from all subjects. All subjects gave written informed consent in accordance with the Declaration of Helsinki. The protocol was approved by the Institutional Review Board of Department of Psychology, Sun Yat-sen University.

## Author Contributions

YL and WL designed the study, collected and analyzed the data, and wrote the manuscript. YY performed the theoretical discussion, analyzed the data, and wrote the manuscript. QW designed the study, analyzed the data, and wrote the manuscript.

## Conflict of Interest Statement

The authors declare that the research was conducted in the absence of any commercial or financial relationships that could be construed as a potential conflict of interest.
